# Effect of the BD132 Dendron Against *Candida tropicalis*: Inhibition of Biofilm Formation and Enzymatic and Structural Alterations

**DOI:** 10.3390/pharmaceutics18050583

**Published:** 2026-05-09

**Authors:** Eloísa García-Porcel, Natalia Gómez-Casanova, Jorge Pérez-Serrano, José Luis Copa-Patiño, Irene Heredero-Bermejo

**Affiliations:** Department of Biomedicine and Biotechnology, Faculty of Pharmacy, Universidad de Alcalá, 28805 Alcalá de Henares, Spain; eloisa.garciap@uah.es (E.G.-P.); natalia.gomezc@uah.es (N.G.-C.); jorge.perez@uah.es (J.P.-S.); josel.copa@uah.es (J.L.C.-P.)

**Keywords:** antifungal resistance, biofilm, *Candida*, membrane disruption, stability, synergy

## Abstract

**Background**: *Candida tropicalis* is a pathogenic yeast species responsible for infections within the *Candida* genus and is identified as the most virulent species after *C. albicans*, partly due to its ability to form biofilms. **Objective**: This study analyzes the antifungal efficacy of a newly synthesized dendron, BD132 dendron, against *C. tropicalis*. **Results**: The compound showed a strong antifungal activity with promising minimum inhibitory concentration (MIC) and minimum biofilm inhibitory concentration (MBIC) values. Combination therapy with AgNO_3_ and amphotericin B showed additive and synergistic effects, respectively, enhancing antifungal efficacy and potentially reducing cytotoxicity. The dendron did not alter key enzyme activities, and scanning electron microscopy revealed significant morphological alterations, including increased cell size and surface damage, indicating membrane disruption. In addition, the BD132 dendron did not induce resistance, and stability studies indicated a slight MIC decrease at 4 °C and −20 °C after 15 days, with stable minimum fungicidal concentration (MFC), suggesting potential for long-term use. **Conclusions**: These findings highlight the potential of this dendron in combination therapies to treat *C. tropicalis* infections.

## 1. Introduction

*Candida* spp. can be part of the human microbiota; however, in immunocompromised patients, hospitalized patients, or people who have undergone surgery, *Candida* spp. can invade tissues, causing a wide range of infections [1,2]. Approximately 400,000 blood infections yearly are caused by *Candida*, with a mortality rate of 40% [3]. These infections are known as candidemia and are common when biofilms are formed on medical devices, such as catheters or implants [4].

Although the majority of infections are related to *C. albicans*, there has been a shift towards non-*albicans Candida* species. This is an important development because many non-*albicans Candida* species show different grades of resistance to the commonly used antifungals [5].

*C. tropicalis* is one of the most frequent non-*albicans Candida* species in infections caused by *Candida* [6], and it is listed in the high-level group of the World Health Organization’s priority list of fungal pathogens [7]. This species shows different virulence factors that allow this pathogen to invade the host’s tissues and evade the immune system. This includes the formation of pseudohyphae, biofilm formation, and secretion of hydrolytic extracellular enzymes like proteinases and phospholipases [8,9].

To date, most in vitro studies with *Candida* have been carried out in planktonic cells; however, the medical impact of *Candida* depends on its ability to form biofilms. These biofilms can grow on implanted medical devices but also on biotic surfaces such as epithelial linings [10]. Moreover, it has been observed that biofilms show 500-fold more resistance to antifungals than planktonic cells [11,12].

To invade host tissues, *C. tropicalis* secretes lytic enzymes such as proteinases, phospholipases and esterases, which disrupt normal host cell function. Proteinases act as a virulence factor by degrading components of the mucosa and immune system [6]. Due to their broad range of substrates—including collagen, keratin and mucin—these enzymes allow *Candida* to break down epithelial barriers as well as immune elements such as antibodies, complement proteins, and cytokines [13]. Phospholipases, which catalyze the hydrolysis of ester bonds in glycerol phospholipids [13], are associated with increased adhesion capacity, greater mortality in animal models, and increased damage to host cell membranes [6]. This effect is particularly significant because phospholipids are major structural components of cell membranes [13]. Additionally, esterases are involved in adhesion to host cells and modulate inflammatory responses by lysing competing microbiota [14].

Related to antimicrobial agents, silver nitrate (AgNO_3_) is one of the most common silver salts used in medicine due to its antibacterial properties, its lack of bacterial resistance, and low toxicity [15]. In addition, AgNO_3_ has shown its effectiveness against *Candida*, probably through the alteration of the membrane integrity [16]. Similarly, amphotericin B is a wide-spectrum drug used in fungal infections. Its mechanism of action involves interaction with the ergosterol in fungal cell membranes, altering their structure and permeability [17]. However, the main problem associated with commonly used drugs is the cytotoxicity and development of resistance. In this sense, combination therapy could be an interesting approach to reduce the required dose of antifungal drugs.

Therefore, *Candida* has become a difficult pathogen to eradicate, highlighting the need for new antifungal compounds. One of the new lines of research is the synthesis of dendritic systems. On the one hand, these molecules show multivalence, which allows them to act as therapeutic agents because of the functional groups on the surface, which may interact with cell membranes [18]. In addition, it is possible to develop specific compounds because of the differences between the cell membranes of fungi and the cell membranes of humans [19]. On the other hand, the solubility of dendritic compounds makes them interesting at a clinical level. Lastly, these compounds could be a solution to the problem of drug resistance because it has been proven that they do not induce resistance in bacteria [18].

The aim of this study is to test the efficacy of a specific dendron against biofilms of *C. tropicalis*. In addition, its activity in combination with AgNO_3_ or with amphotericin B is also evaluated. Moreover, we study the mode of action of the dendritic system by performing an enzymatic test to determine whether the compound affects the secretion of certain enzymes. Finally, the structural alterations produced by the compound on the surface of *C. tropicalis* are studied by scanning electron microscopy.

## 2. Materials and Methods

### 2.1. C. tropicalis: Growing Conditions and Stimulation of Biofilm Formation

The *C. tropicalis* strain used in this study was ATCC 750 (American Type Culture Collection). The *C. tropicalis* isolate was kept at −20 °C with 20% glycerol until use.

The strain was grown overnight on Sabouraud chloramphenicol agar. Then, for experiments that required biofilm formation (Section 2.4 and Section 2.5), colonies were transferred to 45 mL of yeast extract (1%)–peptone (2%)–dextrose (2%) medium (YPD) to stimulate biofilm formation. *C. tropicalis* was incubated with agitation (150 rpm) at 37 °C overnight.

### 2.2. Dendritic Compound, AgNO_3_ and Amphotericin B

**Figure 1:**
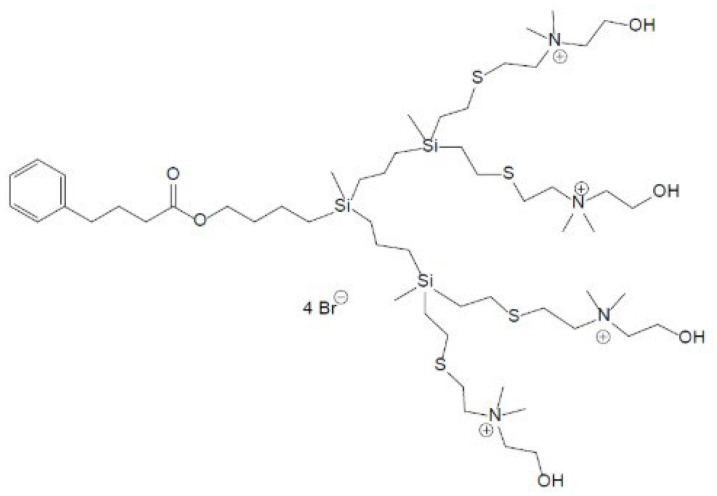
Schematic structure of the BD132 dendron.

The BD132 dendron was tested against *C. tropicalis* to determine its antifungal and antibiofilm capacity. The BD132 dendron (Figure 1) has been previously described [20]. Additionally, we used commercially available AgNO_3_, known for its antimicrobial properties, and amphotericin B, a widely used antifungal agent. The dendron was stored at 4 °C, AgNO_3_ was kept at ambient temperature, and amphotericin B was kept frozen. All three compounds are soluble in water, and the dilutions were prepared on the same day the experiments were performed.

### 2.3. Antifungal Activity Assays on Planktonic Cells

To determine the activity of the BD132 dendron against *C. tropicalis*, the EUCAST standardized protocol was followed [21]. First, the inoculum was adjusted to 0.5 McFarland standard (1 × 10^6^ cells/mL) in distilled water by selecting colonies from a Sabouraud agar plate. After that, the inoculum was diluted to a 1:10 dilution in RPMI-1640 with morpholinepropanesulfonic acid (MOPS, Sigma-Aldrich, Saint Louis, MO, USA) and 2% glucose (Scharlab, Barcelona, Spain) (RPMI-MOPS-GLU).

Then, 100 μL of the suspension was inoculated in 96-well microtiter plates with 100 μL of the BD132 dendron in two-fold serial dilutions ranging from 256 mg/L to 0.25 mg/L. Plates were sealed with Parafilm and incubated for 48 h at 37 °C.

After the incubation time, the minimum inhibitory concentration (MIC), defined as the minimum concentration that inhibits *Candida* growth, was determined by reading the absorbance at 540 nm. Moreover, the minimum fungicidal concentration (MFC), defined as the minimum concentration of the dendron that kills *Candida* cells, was determined using the drop plate method (Section 2.8).

### 2.4. Inhibition of Biofilm Formation

To determine the ability to inhibit biofilm formation, the protocol described in previous studies [18] was followed. First, the inoculum, already grown in YPD, was prepared as described in Section 2.1. Afterwards, the inoculum was adjusted to 0.5 McFarland standard in RPMI-MOPS-GLU.

Then, 50 μL of the suspension was inoculated in a 96-well NUNC^TM^ microtiter plate with 50 μL of the compound (B132 dendron, AgNO_3_ and amphotericin B) in two-fold serial dilutions ranging from 0.5 mg/L to 512 mg/L. Controls without inoculum and without treatment were included in each assay. Plates were sealed with Parafilm and incubated for 48 h at 37 °C. After the incubation time, the minimum biofilm inhibitory concentration (MBIC), defined as the lowest concentration that inhibits biofilm formation, was determined using the resazurin method (Section 2.7), and the minimum fungicidal concentration in biofilm (MFCB), defined as the minimum concentration that kills the cells that are forming a biofilm, was determined using the drop plate method (Section 2.8) [22].

### 2.5. Antifungal Activity Assays on Established Biofilms

To determine the activity of the dendron against previously established biofilms, the method detailed in earlier studies [23] was followed. In this assay, the first step was the formation of the biofilm. For this, 100 μL of an inoculum, previously grown in YPD, at a density of 0.5 McFarland standard in RPMI-MOPS-GLU was transferred into a 96-well NUNC^TM^ microtiter plate (NUNC, Roskilde, Denmark). This plate was sealed with Parafilm and incubated for 48 h at 37 °C.

Then, the medium was removed, and the biofilm was washed with phosphate-buffered saline. Finally, 100 μL of the BD132 dendron dilutions prepared in RPMI-MOPS-GLU (1024 mg/L to 0.5 mg/L) were added. Controls without inoculum and without treatment were included in all experiments. Plates were sealed with Parafilm and incubated for 48 h at 37 °C. Afterwards, to determine the minimum biofilm damaging concentrations (MBDCs), defined as the minimum concentration that inhibits the viability of biofilm-forming cells, the resazurin method was used (Section 2.7), and to determine the minimum biofilm eradicating concentrations (MBECs), defined as the lowest concentration that kills cells that form a biofilm, the drop plate method was used (Section 2.8).

### 2.6. Combination Therapy of the Dendron BD132 and AgNO_3_ or Amphotericin B

Combination therapy tests were performed to evaluate if there was a synergistic effect between the BD132 dendron and other compounds (AgNO_3_ and amphotericin B) against *C. tropicalis.* The checkerboard titration technique [24] was carried out. An inoculum of *Candida* from a YPD culture was adjusted to 0.5 McFarland in RPMI + MOPS + GLU (as described in Section 2.4) and added to a 96-well NUNC^TM^ microtiter plate containing different treatment combinations: 0.25 mg/L to 8 mg/L of BD132 dendron, 0.03 mg/L to 2 mg/L AgNO_3_, and 0.007 mg/L to 0.25 mg/L amphotericin B. Controls without treatment and controls of the MBIC of each compound were included in each experiment. After the incubation period, the resazurin assay and the drop plate method were performed (Section 2.7 and Section 2.8).

To determine the interaction between the dendron and the other compounds, the fractional inhibitory concentration index (FICI) was calculated according to the following equation:

FICI = [(MBIC of BD132 dendron in combination)/(MBIC of BD132 dendron alone)] + [(MBIC of AgNO_3_/amphotericin B in combination)/(MBIC of AgNO_3_/amphotericin B alone)]

The results were considered as follows: synergy: FICI ≤ 0.5; additive: 0.5 < FICI ≤ 1; indifferent: 1 < FICI < 4; and antagonistic: FICI ≥ 4 [24].

Data were represented using GraphPad Prism 10^®^ (GraphPad Software, San Diego, CA, USA).

### 2.7. Resazurin Assay

The resazurin colorimetric assay was used to determine cell viability. A 0.01% resazurin solution was prepared in distilled water. The solution was filtered and kept at 4 °C. After treatment, wells were washed with PBS, and 20 μL of resazurin and 100 μL of PBS were added. Then, plates were incubated for 24 h at 37 °C in the dark, and absorbance was measured at 570 nm using a microplate reader. This method was used to determine the MBIC values in inhibition of biofilm formation experiments (Section 2.4) and the MBDC values in established biofilm experiments (Section 2.5).

### 2.8. Drop Plate Method

The drop plate method was used to determine the MFC values in planktonic cells (Section 2.3), the MFCB values in the inhibition of biofilm formation experiments (Section 2.4), and the MBEC values in established biofilm experiments (Section 2.5).

Biofilms were scraped and suspended, and a 5 μL aliquot was transferred onto Sabouraud chloramphenicol agar plates. The plates were incubated for 24 h at 37 °C.

### 2.9. Determination of Alterations in Enzymatic Activity

To determine the effect of the BD132 dendron on the enzymatic activity of *C. tropicalis* biofilm cells, the following assays were performed. The enzymes studied were aspartyl proteinase (Sap), phospholipase and esterase. The enzymatic activity was determined by inoculating the plates with 5 μL from microtiter plates of the inhibition of biofilm formation experiments (Section 2.4). The concentrations tested were the MIC and three concentrations below it, as well as a control without treatment in all experiments.

After the incubation time, the colony and the clear zone were measured in order to determine the enzyme activity. The Pz was calculated as follows:

Pz = diameter of the colony/diameter of the colony plus the clear zone.

Absence of enzymatic activity was defined as a value of 1, low enzymatic activity as 0.700 ≤ Pz ≤ 0.999, moderate activity as 0.400 ≤ Pz ≤ 0.699, and high enzymatic activity as 0.100 ≤ Pz ≤ 0.399 [8].

#### 2.9.1. Aspartyl Proteinase (Sap)

To test the activity of the secreted Sap, we used a medium described by Galán-Ladero [25]. First, we sterilized 225 mL of distilled water containing 5 g of agar. Second, we prepared a solution that contained 2.92 g of yeast carbon base, 0.025 g of yeast extract, and 0.5 g of bovine serum albumin (BSA) in 25 mL of distilled water and a pH of 5. This solution was sterilized by filtration with a 0.2 µm filter and added to the autoclaved water containing 5 g of agar.

To determine the changes in enzymatic activity, 5 µL from each well of the plate was transferred to the Petri dishes containing this medium. The plates were incubated for 7 days at 37 °C. Then, the plates were prepared as described by El-kholy [8]. First, the colonies were fixed using 20% trichloroacetic acid. Then, the plates were stained with 1.25% amido black dye and destained with 30% acetic acid. The cleared zone produced by the cleavage of BSA was measured, and proteinase activity was determined by calculating the Pz value.

#### 2.9.2. Phospholipase

To assess phospholipase activity, we prepared a specific medium described by Galán-Ladero [25]. The medium contains Sabouraud chloramphenicol agar with 3% glucose, 1 M NaCl and 0.005 M CaCl_2_. The solution was autoclaved and supplemented with 5% sterile egg yolk emulsion.

A 5 µL aliquot from the microtiter plates was inoculated on the agar Petri dishes and incubated for 7 days at 37 °C. During this time, the phospholipase activity produced a precipitation zone around the colony, which was measured.

#### 2.9.3. Esterase

Finally, to study the esterase activity, the protocol detailed in Galán-Ladero [25] was performed. Plates were prepared with a medium containing 2.5 g of Bacto Peptone, 1.25 g of NaCl, 0.025 g of CaCl_2_ and 5 g of agar in distilled water. The solution was autoclaved, and 1.25 mL of 80% Tween was added.

Finally, a 5 µL aliquot was inoculated onto the Petri dish and incubated for 7 days at 37 °C. After this time, the precipitation zone formed by the release of fatty acids bound to the calcium in the medium was measured.

### 2.10. Antifungal Resistance Induction on C. tropicalis

To evaluate the induction of resistance caused by the BD132 dendron in *C. tropicalis*, we performed a study to monitor the MIC value daily for 15 days based on the protocol described by Fuentes-Paniagua [26] with slight modifications to adapt it for *Candida*. The inoculum was prepared as described for the planktonic cell assays (Section 2.3), adjusting to 0.5 McFarland (1 × 10^6^ cells/mL) in distilled water and diluting at 1:10 in RPMI + MOPS + GLU. Then, 1 mL of this inoculum was added to 1 mL of the dendron concentrations. The tested concentrations were the MIC value, one concentration higher, and one concentration lower. A control without the compound was included in all experiments. The tubes were incubated for 24 h at 37 °C, and the absorbance was measured at 540 nm. The second day, a new inoculum adjustment was performed from the previous tube, adjusting to 0.5 McFarland, diluting it, and mixing it with the dendron. This process was repeated daily for 15 cycles, analyzing the MIC by adding dendron every day to the *C. tropicalis* culture.

### 2.11. Stability of the Compound

The stability of the compounds stored in water was studied to determine the optimal storage conditions. Stability of the compound was evaluated over 15 days. For this purpose, two conditions were established: 4 °C (refrigerator) and −20 °C (freezer). To perform the assay, the protocol for determining the MIC in planktonic cells (Section 2.3) was used. On the first day of the assay, different concentrations of the diluted compound were stored at both 4 °C and −20 °C. In the following days, the assay was run using these prediluted dendron aliquots stored at different temperatures instead of preparing new dilutions. In the case of the solution at −20 °C, it was necessary to freeze and thaw the dilutions each day. The last assay was performed 15 days after the dilutions were prepared.

### 2.12. Structural Study

Cellular damage produced by the BD132 dendron in *C. tropicalis* biofilms was evaluated using scanning electron microscopy (SEM). Sublethal concentrations, inhibitory concentrations, and controls were tested.

*C. tropicalis* was cultured on a glass coverslip following the procedure described previously (Section 2.4). After incubation and treatment, samples were fixed with a Milloning’s 2% glutaraldehyde solution for 24 h. Then, coverslips were washed in Milloning’s solution and dehydrated using ethanol (30%, 50%, 70%, 95%, and 100%), with 7 min incubation for each concentration. Then, coverslips were kept in anhydrous acetone. The samples were processed using a critical-point drying system and coated with a 200 Å gold–palladium layer using a Polaron E5400 sputter coater (Quorum Technologies. Laughton, UK). Scanning electron microscopy (SEM) was conducted with a Jeol JSM-IT500 microscope (JEOL Ltd. Akishima, Tokyo, Japan) operating at 5–15 kV. Multiple fields were examined for each sample.

### 2.13. Statistical Analysis

All experiments in this study were performed in triplicate, with three replicates conducted in each trial. Statistical analysis was conducted using Microsoft^®^ Excel^®^ for Microsoft 365 MSO (version 2410). The effect of the dendron on the percentage of viability was studied using one-way ANOVA in GraphPad Prism 10^®^ (GraphPad Software, San Diego, CA, USA), and the graphical representations were generated using the same program.

## 3. Results and Discussion

### 3.1. Antifungal Activity of the BD132 Dendron Against Planktonic and Biofilms of C. tropicalis

**Figure 2:**
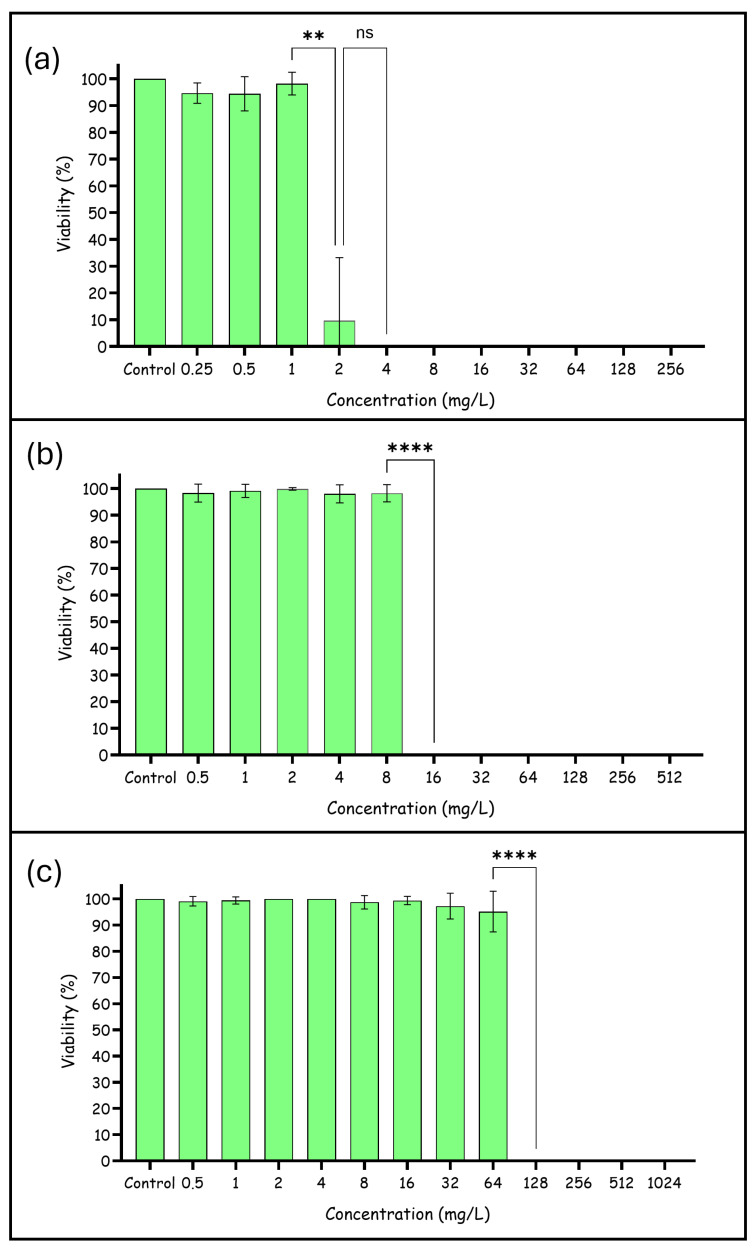
Effect of the dendron BD132 against *C. tropicalis*: (**a**) planktonic cells, (**b**) inhibition of biofilm formation, (**c**) eradication of a pre-formed biofilm. ns (non-significant differences), ** (*p*-value < 0.01), **** (*p*-value < 0.0001).

The result obtained showed that the BD132 dendron inhibited *C. tropicalis* growth at 2–4 mg/L (MIC) (Figure 2), while the MFC value was 4 mg/L (Table 1). In assays of biofilm formation inhibition, the MBIC and MFCB values were 16 mg/L. However, the concentration of BD132 dendron against established biofilms was higher; the MBDC was 128 mg/L (Figure 2), and the MBEC was 1024 mg/L (Table 1).

In view of the results obtained, the dendron tested is promising, as the MIC and MBIC values are not toxic to human cells (low toxicity at 32 mg/L and not toxic at 16 mg/L [20]). However, the concentration of dendron corresponding to MBDC showed toxicity against human cells. This fact is relatively common in antifungal therapy, as many of the available antifungal agents at effective concentrations are toxic to humans, such as amphotericin B [17].

### 3.2. Combined Activity of BD132 Dendron and Commercial Antifungals

The activity of the BD132 dendron in combination with two different antimicrobials was studied (AgNO_3_ and amphotericin B). The study was performed under biofilm formation inhibition conditions.

The results showed an MBIC of 8 mg/L for BD132 dendron (a reduction from 16 mg/L for the BD132 dendron alone) when combined with 0.03 mg/L of AgNO_3_ (a reduction from 2 mg/L alone). Furthermore, the results obtained for the MFCB of the combination of the two compounds were the same as the MBIC (results shown in Table 2). This combination showed an additive effect in inhibiting and killing biofilm formation. Therefore, these results reveal that the combined use of AgNO_3_ and BD132 dendron enhances the antifungal efficacy of both compounds compared with their individual use. AgNO_3_ demonstrated a disruptive effect on the *Candida* membrane and an increase in its permeability. Additionally, a reduction in ATPase levels in fungal cells treated with AgNO_3_ was observed, which may indicate an interaction with ATPase or an increase in oxidative stress [27]. By combining AgNO_3_ with the dendron, it is possible that each compound acts differently, enhancing the overall efficacy of the treatment with a lower dose of each compound than would be required if used individually.

On the other hand, the results obtained for MBIC with the combination of the BD132 dendron and amphotericin B showed synergy at five different concentrations, as shown in Table 3. In this respect, it is remarkable that the synergy effect of the combination of 4 mg/L of BD132 dendron with 0.06 mg/L of amphotericin B obtained a reduction in the dendron from 16 mg/L to 4 mg/L and a reduction in amphotericin B from 0.5 mg/L to 0.06 mg/L. The combination of 0.25 mg/L of BD132 dendron and 0.12 mg/L of amphotericin B is also interesting, as this combination showed a higher reduction in the concentration of BD132 dendron (64-fold reduction) but a lower reduction in amphotericin B (16-fold reduction) than the combination mentioned above.

Furthermore, related to the MFCB, the combination of the dendron with amphotericin B showed synergy. Combining 4 mg/L of the dendron with 0.12 mg/L of amphotericin B resulted in a reduction from 16 mg/L to 4 mg/L for the BD132 dendron and from 0.5 mg/L to 0.12 mg/L for amphotericin B. However, the other combinations showed an additive effect, as shown in Table 4.

The combination of the dendron with amphotericin B showed a remarkable synergistic result. This means that the two compounds in combination are more effective in inhibiting the biofilm formation of *C. tropicalis* than the compounds when used individually. Amphotericin B primarily works by binding to ergosterol in the fungal cell membrane, altering its permeability. However, fungal cells have many similarities to human cells, which means that many of the drugs are cytotoxic. A clear example of this is amphotericin B, which interacts with cholesterol present in the membranes of our cells, causing nephrotoxicity and cardiotoxicity [17].

The additive and synergistic effects shown by these combined therapies might be of great clinical relevance by achieving a reduction in the effective doses of each compound, thereby reducing the risk of side effects and cytotoxicity. This approach also establishes itself as a strategy to overcome antifungal resistance, which is particularly important in the context of fungal infections where biofilms play a critical role in treatment resistance.

### 3.3. Alterations in Enzyme Production

The results obtained showed no enzymatic changes because the Pz value did not change when the dendron was used. On the one hand, the strain did not show phospholipase activity. On the other hand, the strain had moderate esterase activity and low proteinase activity. Therefore, the treatment with the BD132 dendron did not produce any alteration in enzyme production (Table 5), which is interesting because proteinases and esterases are enzymes that play a crucial role in the pathogenesis of *Candida*.

### 3.4. Antifungal Resistance Induction in C. tropicalis by BD132 Dendron

The results obtained showed that the dendron did not induce resistance: the MIC values remained stable at 2–4 mg/L during 15 cycles. However, a slight increase in the MFC values was observed (from 4 mg/L to 4–8 mg/L), although the *C. tropicalis* cells were successfully eradicated. The stability of the MIC suggests that *C. tropicalis* does not develop resistance to the dendron even after prolonged exposure. This is an interesting finding, as one of the major challenges in treating fungal infections is the emergence of strains resistant to commercial antifungals.

In comparison to many conventional antifungals, in cases where resistance development has been observed—especially with continuous or prolonged use—the absence of resistance to the dendron highlights its potential as a sustainable long-term therapeutic option. The stability of the MIC supports the viability of the dendron in combination therapies. Its combined use with other antifungals, such as amphotericin B or AgNO_3_, can not only enhance efficacy but also reduce the risk of developing resistance to both compounds.

These results may be related to the fact that the dendron did not affect the enzymatic activity of *C. tropicalis*, as many antifungals induce pressure on fungal cells that leads to the activation of resistance mechanisms. For example, subinhibitory concentrations of fluconazole induce higher secretion of proteinases in *Candida* biofilms [28]. In addition, a relationship between biofilm formation, proteinase prevalence, and resistance in *Candida* cells [29] was observed, which may suggest that one reason the dendron does not induce resistance is that it may not trigger proteinase production.

### 3.5. Stability of the BD132 Dendron Under Storage Conditions

The stability study of the BD132 dendron in aqueous solution under different storage temperatures (4 °C and −20 °C) during 15 days produced valuable findings. At both temperatures, a slight decay in compound activity was observed. This was reflected by an increase in the MIC values, which rose from 2–4 mg/L to 4 mg/L. This change in the MIC occurred on day 13 when solutions were stored at 4 °C and on day 7 when solutions were stored at −20 °C and was maintained until day 15. However, it is important to note that the MFC values remained stable at 4 mg/L throughout the storage period under both conditions.

These findings are promising, as the stability of the MFC suggests that the compound retains its fungicidal properties over time, even if its inhibitory concentration slightly increases. This is crucial for ensuring the effectiveness of the compound in long-term storage and potential clinical applications.

The observation that the compound deteriorates faster at −20 °C than at 4 °C could be attributed to repeated freeze–thaw cycles. The process of freezing and thawing can lead to physical and chemical changes in the compound, potentially affecting its stability.

### 3.6. Structural Alterations in C. tropicalis Biofilms

**Figure 3:**
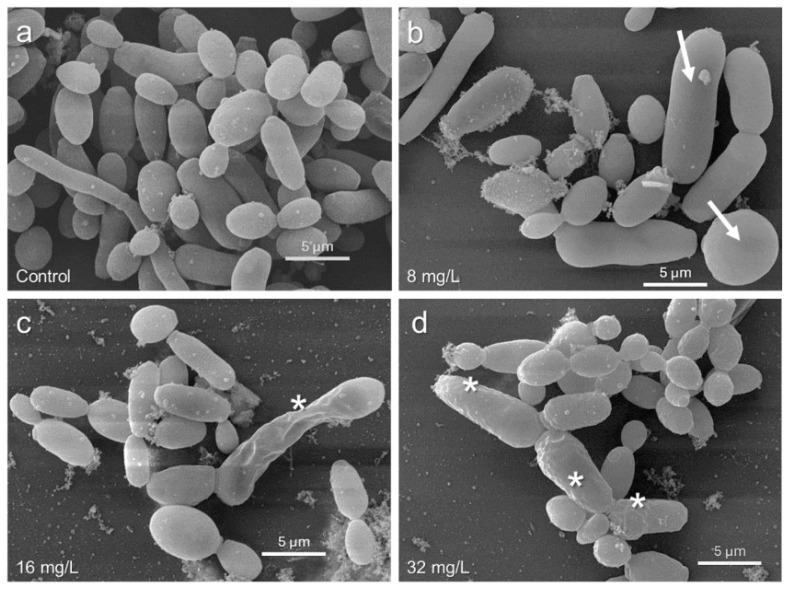
SEM images of the effect of the BD132 dendron on inhibiting biofilm formation: (**a**) Control without treatment; (**b**) *C. tropicalis* treated with 8 mg/L; (**c**) Cells treated with the MBIC (16 mg/L); (**d**) *Candida* cells treated with 32 mg/L. Cell damage (*), swollen cells (→).

SEM studies showed changes in the cell morphology of *C. tropicalis* after treatment with the BD132 dendron (Figure 3). In the control samples, a well-formed biofilm with the characteristic hyphal structures of *C. tropicalis* was observed (Figure 3a). Hyphae are crucial for the formation and maintenance of the biofilm, providing a robust structural framework that facilitates resistance to antifungals and host immune response.

In samples treated with low dendron concentrations, a morphological alteration in the *Candida* cells was observed, presenting a smooth surface but a larger size compared to the control (Figure 3b).

Finally, with increasing dendron concentration, significant damage to the cell surface, including aberrations and perforations (Figure 3c,d), was observed.

SEM images provided visual evidence of the effects of the dendron on *C. tropicalis*. The observation of damaged cell surfaces and reduced cell density corroborates the quantitative results obtained in the biofilm inhibition and eradication assays. Finally, alterations in cell morphology, such as increased size (Figure 3b) and surface damage (Figure 3c,d), suggest that the dendron may be interfering with processes crucial for cell integrity.

## 4. Conclusions

The newly synthesized dendron (BD132) shows promising potential in treating *C. tropicalis* infections. It exhibited strong antifungal activity with low MIC and MBIC values that were non-toxic to human cells, although the MBDC concentration was cytotoxic. This issue could be mitigated by using combination therapies to reduce effective dosages and, consequently, cytotoxicity. In this context, the dendron demonstrated additive effects in combination with AgNO_3_ and synergistic effects with amphotericin B in the inhibition of biofilm formation, enhancing the overall antifungal efficacy of both compounds.

The BD132 dendron did not affect key enzyme activities; moreover, it did not induce resistance in planktonic cells, making it advantageous over conventional antifungals. The compound demonstrated good long-term stability when stored properly. Additionally, morphological changes observed via SEM showed larger, smooth-surfaced cells at low concentrations and significant cell surface damage at higher concentrations.

Overall, the dendron offers a promising approach for the treatment of *C. tropicalis* infections, particularly in combination therapies, to enhance efficacy and reduce cytotoxicity and resistance development. Future studies should optimize storage conditions, explore combination therapies, and investigate the effect of the BD132 dendron on pre-formed biofilms.

## Figures and Tables

**Table 1:** Activity of BD132 dendron against *C. tropicalis*. Concentrations expressed in mg/L.

Planktonic Cells		Biofilm Formation		Established Biofilm	
MIC *	MFC *	MBIC *	MFCB *	MBDC *	MBEC *
2–4	4	16	16	128	1024

* MIC: minimum inhibitory concentration; MFC: minimum fungicidal concentration; MBIC: minimum biofilm inhibitory concentration; MFCB: minimum fungicidal concentration in biofilm; MBDC: minimum biofilm damaging concentration; MBEC: minimum biofilm eradicating concentration.

**Table 2:** Results obtained by combining BD132 dendron with AgNO_3_.

MBICs and MFCBs *					
BD132 Dendron Individual	AgNO_3_ Individual	BD132 Dendron + AgNO_3_		FICI **	Effect
		BD132 Dendron	AgNO_3_		
16 mg/L	2 mg/L	8 mg/L	0.03 mg/L	0.515	Additive

* MBIC: minimum biofilm inhibitory concentration; MFCB: minimum fungicidal concentration in biofilm; FICI: fractional inhibitory concentration index. ** FICI = [(MBIC of BD132 dendron in combination)/(MBIC of BD132 dendron alone)] + [(MBIC of AgNO_3_ in combination)/(MBIC of AgNO_3_ alone)]. Synergy FICI ≤ 0.5; additive 0.5 < FICI ≤ 1; indifferent 1 < FICI < 4; antagonistic FICI ≥ 4.

**Table 3:** MBIC results obtained for the combination of BD132 dendron and amphotericin B.

MBICs *					
BD132 Dendron Individual	AmpB Individual	BD132 Dendron + AmpB *		FICI **	Effect
		BD132 Dendron	AmpB		
16 mg/L	0.5 mg/L	8 mg/L	0.007 mg/L	0.514	Additive
		4 mg/L	0.06 mg/L	0.370	Synergy
		2 mg/L	0.12 mg/L	0.365	Synergy
		1 mg/L	0.12 mg/L	0.302	Synergy
		0.5 mg/L	0.12 mg/L	0.271	Synergy
		0.25 mg/L	0.12 mg/L	0.255	Synergy

* MBIC: minimum biofilm inhibitory concentration; AmpB: amphotericin B; FICI: fractional inhibitory concentration index. ** FICI = [(MBIC of BD132 dendron in combination)/(MBIC of BD132 dendron alone)] + [(MBIC of amphotericin B in combination)/(MBIC of amphotericin B alone)]. Synergy FICI ≤ 0.5; additive 0.5 < FICI ≤ 1; indifferent 1 < FICI < 4; antagonistic FICI ≥ 4.

**Table 4:** MFCBs results obtained for the combination of BD132 dendron and amphotericin B.

MFCBs *					
BD132 Dendron Individual	AmpB Individual	BD132 Dendron + AmpB *		FICI **	Effect
		BD132 Dendron	AmpB		
16 mg/L	0.5 mg/L	8 mg/L	0.01 mg/L	0.520	Additive
		4 mg/L	0.12 mg/L	0.490	Synergy
		2 mg/L	0.25 mg/L	0.625	Additive
		1 mg/L	0.25 mg/L	0.562	Additive
		0.5 mg/L	0.25 mg/L	0.531	Additive
		0.25 mg/L	0.25 mg/L	0.515	Additive

* MFCB: minimum fungicidal concentration in biofilm; AmpB: amphotericin B; FICI: fractional inhibitory concentration index. ** FICI = [(MFCB of BD132 dendron in combination)/(MFCB of BD132 dendron alone)] + [(MFCB of amphotericin B in combination)/(MFCB of amphotericin B alone)]. Synergy FICI ≤ 0.5; additive 0.5 < FICI ≤ 1; indifferent 1 < FICI < 4; antagonistic FICI ≥ 4.

**Table 5:** Enzymatic activity of *C. tropicalis* 7 days after treatment with BD132 dendron.

Enzyme	Control	BD132 Dendron Treatment		
		8 mg/L	4 mg/L	2 mg/L
Esterase	Medium	Medium	Medium	Medium
Aspartyl proteinase	Low	Low	Low	Very low
Phospholipase	None	None	None	None

Pz = diameter of the colony/diameter of the colony plus the clear zone. Pz = 1 (no enzymatic activity); 0.700 ≤ Pz ≤ 0.999 (low enzymatic activity); 0.400 ≤ Pz ≤ 0.699 (medium activity).

## Data Availability

The raw data supporting the information on this manuscript will be made available by the authors on request.

## Abbreviations

AmpB	Amphotericin B
BSA	Bovine Serum Albumin
FICI	Fractional Inhibitory Concentration Index
MBDC	Minimum Biofilm Damaging Concentration
MBEC	Minimum Biofilm Eradicating Concentration
MBIC	Minimum Biofilm Inhibitory Concentration
MFC	Minimum Fungicidal Concentration
MFCB	Minimum Fungicidal Concentration in Biofilm
MIC	Minimum Inhibitory Concentration
Sap	Aspartyl Proteinase
SEM	Scanning Electron Microscopy
YPD	Yeast Extract (1%)–Peptone (2%)–Dextrose (2%)
